# Impact of Transmammary-Delivered Meloxicam on Biomarkers of Pain and Distress in Piglets after Castration and Tail Docking

**DOI:** 10.1371/journal.pone.0113678

**Published:** 2014-12-01

**Authors:** Jessica L. Bates, Locke A. Karriker, Matthew L. Stock, Kelly M. Pertzborn, Luke G. Baldwin, Larry W. Wulf, C. J. Lee, Chong Wang, Johann F. Coetzee

**Affiliations:** 1 Swine Medicine Education Center, Veterinary Diagnostic and Production Animal Medicine, College of Veterinary Medicine, Iowa State University, Ames, Iowa, United States of America; 2 Pharmacology Analytical Support Team, Veterinary Diagnostic and Production Animal Medicine, College of Veterinary Medicine, Iowa State University, Ames, Iowa, United States of America; 3 Veterinary Diagnostic and Production Animal Medicine, College of Veterinary Medicine, Iowa State University, Ames, Iowa, United States of America; University of Bari, Italy

## Abstract

To investigate a novel route for providing analgesia to processed piglets via transmammary drug delivery, meloxicam was administered orally to sows after farrowing. The objectives of the study were to demonstrate meloxicam transfer from sows to piglets via milk and to describe the analgesic effects in piglets after processing through assessment of pain biomarkers and infrared thermography (IRT). Ten sows received either meloxicam (30 mg/kg) (n = 5) or whey protein (placebo) (n = 5) in their daily feedings, starting four days after farrowing and continuing for three consecutive days. During this period, blood and milk samples were collected at 12-hour intervals. On Day 5 after farrowing, three boars and three gilts from each litter were castrated or sham castrated, tail docked, and administered an iron injection. Piglet blood samples were collected immediately before processing and at predetermined times over an 84-hour period. IRT images were captured at each piglet blood collection point. Plasma was tested to confirm meloxicam concentrations using a validated high-performance liquid chromatography-mass spectrometry method. Meloxicam was detected in all piglets nursing on medicated sows at each time point, and the mean (± standard error of the mean) meloxicam concentration at castration was 568.9±105.8 ng/mL. Furthermore, *ex-vivo* prostaglandin E_2_ (PGE_2_) synthesis inhibition was greater in piglets from treated sows compared to controls (p = 0.0059). There was a time-by-treatment interaction for plasma cortisol (p = 0.0009), with meloxicam-treated piglets demonstrating lower cortisol concentrations than control piglets for 10 hours after castration. No differences in mean plasma substance P concentrations between treatment groups were observed (p = 0.67). Lower cranial skin temperatures on IRT were observed in placebo compared to meloxicam-treated piglets (p = 0.015). This study demonstrates the successful transfer of meloxicam from sows to piglets through milk and corresponding analgesia after processing, as evidenced by a decrease in cortisol and PGE_2_ levels and maintenance of cranial skin temperature.

## Introduction

Pork producers and consumers are increasingly concerned about the well-being of food producing animals. The management of pain during routine swine husbandry practices, such as castration and tail docking in piglets, is of particular significance. The European Union (EU) recently moved to ensure that all piglets are castrated using analgesia/anesthesia [1]. However, in the United States, there are currently no Food and Drug Administration (FDA)-approved drug regimens for pain relief in livestock, and analgesia is not routinely provided at the time of processing.

Meloxicam is a non-steroidal anti-inflammatory drug (NSAID) that is approved for swine in the EU and Canada for several conditions, including the relief of post-operative pain with minor soft tissue surgery. When injected before piglet castration, meloxicam reduces serum cortisol concentrations [2], [3], [4]. Meloxicam has also been shown to reduce behavioral signs that are associated with piglet distress at castration and is considered to be superior to other analgesics when assessing pain-related behavioral criteria [5].

Administering oral meloxicam to sows during lactation would potentially provide analgesia during processing procedures by allowing passive drug transfer through the milk to entire litters. This route is safer for both the handler and the animal when compared to injections. It is also easily administered and allows a large number of animals to be medicated, thus eliminating the need for individual injections. Although there are no peer-reviewed studies demonstrating transmammary analgesia in swine, NSAIDs can transfer through milk in both cattle [6] and humans [7], [8], [9]. The objectives of this study were to demonstrate the transmammary delivery of meloxicam from sows to piglets and to assess the pharmacodynamics and analgesic effects in piglets after castration. The findings of this study demonstrate the successful transfer of meloxicam from sows to piglets through milk and associated analgesia after processing, as evidenced by a decrease in cortisol and PGE_2_ levels and maintenance of cranial skin temperature.

## Materials and Methods

Before the initiation of this study, all techniques regarding animal use, housing, handling, and sampling were approved by the Iowa State University Animal Care and Use Committee (IACUC # 8-12-7430-S).

### Animals

Ten Yorkshire x Landrace sows at approximately one week prior to farrowing (average weight of 277.3 kg) were obtained from a commercial swine farm. Upon arrival, each sow was confirmed to be healthy and pregnant by a veterinarian, and a unique numerical ear tag (Allflex Global Ear Tags, Allflex USA, Inc., DFW Airport, TX) was applied to the right ear. Sows were housed at the Iowa State University Animal Resource Station in accordance with recommendations outlined in the Guide for the Care and Use of Agricultural Animals in Agricultural Use and Research and Teaching [10]. Sows were placed in Quad- or Euro-style farrowing stalls (Thorp Equipment, Thorp, WI), depending on availability. Both stall types were equally represented in both treatment groups. Regardless of stall type, each sow was housed in a farrowing stall area measuring 0.6 m×2.1 m. Quad and Euro stalls had piglet creep areas of 7.0 m^2^ and 6.4 m^2^, respectively. After farrowing, a heat lamp was provided on each side of the creep area in each stall for the piglets.

### Feeding and Treatment Administration

Prepartum sows were hand-fed 1.6 kg of an organic corn/soybean meal diet, which was confirmed to be free of meloxicam, twice daily. This diet was compatible with the National Research Council's nutrient requirements for lactating sows [11]. Intake was gradually increased ad-libitum after farrowing. Sows had free access to water at all times through a nipple waterer in their stalls. On Day 4 after farrowing, sow treatments began and continued for six days. Sows assigned to the meloxicam-treatment group (n = 5) received 30 mg/kg meloxicam (Meloxicam, Aurobindo Pharma, India, Batch X1513019-A, Expiration Date 2/2015), which was divided between 2 feedings at 0700 h and 1600 h. The meloxicam was ground from tablets into a powdered consistency using a commercial grinder (Spice & Nut Grinder, Cuisinart, East Windsor, NJ), after which it was incorporated into each sow's daily feed ration in a portable mixer (Kobalt Model #043206, Monarch Industries, Winnipeg, MB, Canada). Control sows (n = 5) received 30 mg/kg of whey protein placebo (Health Watchers, Inc., Bohemia, NY), which is a pharmacologically inactive excipient used in the manufacturing of meloxicam tablets. The placebo was prepared in a separately marked bucket by thorough hand-mixing with gloved hands to prevent cross contamination.

### Animal phase study design

**Figure 1:**
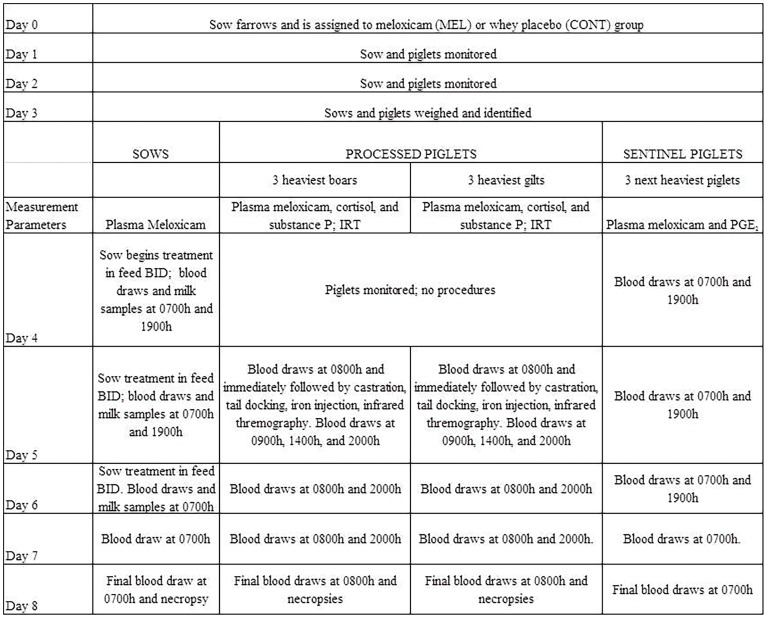
Outline of study events for sows and their litters.

The sows were allowed to farrow naturally without induction methods. They were then randomly assigned to two groups. The first sow to give birth was randomly allocated to the meloxicam-treated group (MEL), and the second sow was allocated to the whey placebo group (CONT). The alternating pattern continued for the remaining sows, based on the farrowing date. The day of farrowing was designated as “Day 0” for each sow and litter. The overall time scheme of activities, including drug administration and sample collection, is detailed in Figure 1.

On Day 3, post-farrowing piglets in the litter were weighed and ranked in a descending order. In each litter, the heaviest three boars and three gilts were selected and ear tagged (Allflex Global Ear Tags, Allflex USA, Inc., DFW Airport, TX). The next three heaviest piglets, regardless of sex, were selected and tagged as sentinels to specifically measure the inhibition of plasma prostaglandin E_2_ (PGE_2_) levels and demonstrate the pharmacodynamic effect of meloxicam. In total, nine piglets per litter were tagged. Two litters did not have three live boar piglets. In those instances, all available boars were used as test piglets. In another litter, a male test piglet was laid on and subsequently died after being identified but before blood sampling began. No other piglets were substituted into the test category. Cross-fostering was not performed at any phase of the study.

Piglet processing occurred on Day 5 after farrowing. After a pre-processing blood draw, the boars were immediately castrated and tail-docked. They then received 1 mL (100 mg) iron IM (Ferrodex 100, AgriLabs, St. Joseph, MO). Castration was performed in accordance with standard swine industry practices by making two vertical incisions approximately 2-3 cm long in the scrotum with a number-ten scalpel blade and scalpel handle, marsupializing the testicles, and finally providing manual pressure on the spermatic cord until it separated from the piglet's body. Immediately after each piglet castration, the scalpel blade and handle were immersed in a dilute chlorhexidine mixture for disinfection between each piglet procedure per typical swine industry practice. All castrations were performed by a single experienced veterinarian to minimize variation (JLB). Gilts were handled in a similar manner, and they also underwent tail docking and received iron.

Sow blood samples (8 mL/sample) were collected via the left or right jugular vein using a 25.4-mm, 16-gauge hypodermic needle (Air-Tite Products, Virginia Beach, VA) and 12-mL Luer lock syringe (TycoHealth Care, Mansfield, MA). During blood collection, sows were manually restrained in their crates using a pig snare.

Piglet blood samples (2 mL/sample) were collected using the left or right jugular vein using a 3.8-cm, 22-gauge hypodermic needle (TycoHealth Care, Mansfield, MA) and 3-mL syringe (TycoHealth Care, Mansfield, MA). These samples were obtained using physical restraint by placing the piglet in a supine position.

On Day 8 after farrowing, sows were euthanized by a penetrating captive bolt, followed by exsanguination, and piglets were euthanized by blunt force trauma to the cranium, according to American Veterinary Medical Association guidelines [12]. Necropsies were performed on the sows and processed piglets. The liver, kidney, gastric fundus, duodenum, semitendinosus/semimembranosus muscle, and fat were collected for analysis.

### Infrared Thermography

**Figure 2:**
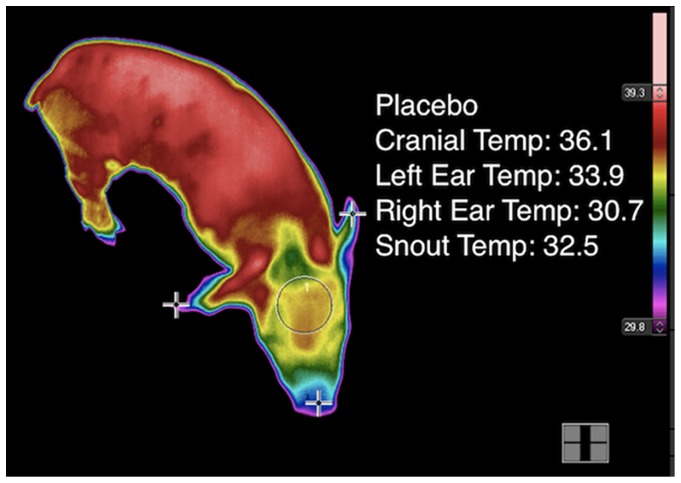
Example of a digital image of infrared thermography (IRT) measurement. Each processed piglet was measured for temperature in °C at the top of the cranium (circled), right and left ears, and snout (cross marks).

Following processing and each blood-sampling time point, changes in piglet skin temperature were measured using a commercially available infrared thermography (IRT) camera (FLIR SC660, Systems, Wilsonville, OR). Prior to each use, the camera was allowed to self-calibrate with the ambient temperature and relative humidity in the barn. Piglets were placed in a non-restrictive plastic tub measuring 50.8 cm in diameter and 43.2 cm tall for approximately ten seconds while thermographic images of the cranium, right and left ears, and snout were obtained (Figure 2).

### Sample Collection, Processing, and Analysis

All drug concentrations in plasma were analyzed at the Iowa State University Veterinary Diagnostic Laboratory by the Iowa State University-Pharmacology Analytical Support Team (ISU-PhAST).

#### Meloxicam Analysis

Blood for meloxicam analysis was placed in 10-mL and 3-mL heparinized blood collection tubes (BD Vacutainer, Franklin Lakes, NJ). Samples were centrifuged for 15 minutes at 1000 g at ambient temperature. The plasma was separated and placed into cryovials for storage at −80°C.

Plasma concentrations of meloxicam were determined using high-performance liquid chromatography (HPLC) (Accela Pump and Autosampler, Thermo Scientific, San Jose, CA, USA) with mass spectrometry (MS) detection (LTQ XL, Thermo Scientific, San Jose, CA, USA). Plasma samples, plasma spikes, and plasma quality control (QC) samples (200 µL each) were treated with 1 M trichloroacetic acid (100 µL) after the addition of the internal standard, piroxicam (10 µL of 10 ng/µL). The samples were vortexed for 5 seconds and centrifuged for 20 minutes at 2000 g to sediment the precipitate. A portion of the supernatant (150 µL) was transferred to an injection vial that was fitted with a glass insert containing 100 µL of 1.9% ammonium hydroxide in 25% aqueous acetonitrile. The injection volume was set to 20 µL. The mobile phases consisted of 0.1% formic acid in water (A) and 0.1% formic acid in acetonitrile (B) at a flow rate of 0.250 mL/min. The mobile phase began at 40% B with a linear gradient to 95% B at 4 minutes, which was maintained for 1.5 minutes, followed by re-equilibration to 40% B. Separation was achieved with a solid-core C18 column (KinetexXB -C18, 100 mm×2.1 mm, 2.6-µm particles, Phenomenex, Torrance, CA, USA) that was maintained at 45°C. Piroxicam eluted at 2.6 minutes, and meloxicam eluted at 3.3 minutes. A full scan MS of the pseudomolecular ions of piroxicam (m/z 332) and meloxicam (m/z 352) was used for analyte detection. The sum of the intensities of ions at m/z of 115 and 141 were used for meloxicam quantitation. The internal standard, piroxicam, was quantitated with the sum of the ion intensities at m/z of 95, 121, and 164. Sequences consisting of plasma blanks, calibration spikes, QC samples, and porcine plasma samples were processed in batches with a processing method that was developed in the Xcalibur software (Thermo Scientific, San Jose, CA, USA). The processing method automatically identified and integrated each peak in each sample and calculated the calibration curve based on a weighted (1/X) linear fit. Plasma concentrations of meloxicam in unknown samples were calculated by the Xcalibur software based on the calibration curve. Results were then viewed in the Quan Browser portion of the Xcalibur software. Fourteen calibration spikes were prepared in porcine plasma covering the concentration range of 1–20,000 ng/mL. QC samples were prepared at concentrations of 15, 150, and 1500 ng/mL in duplicate with each set of samples. Calibration curves exhibited a correlation coefficient (R^2^) exceeding 0.997 across the entire concentration range. The QC samples at 150 and 1500 ng/mL were within 2–8% of their nominal values, and the low QC sample at 15 ng/mL differed from its nominal value by 10–15%.

#### PGE2 Analysis

PGE_2_ concentrations were determined using methods that were previously described [13]. Briefly, fresh piglet blood was collected into sterile tubes containing heparin. To stimulate ex-vivo PGE_2_ production by monocytes, the heparinized whole blood was incubated for 24 hours at 37°C with 10 µg/ml lipopolysaccharide (LPS, derived from *Escherichia coli* 055:B5, Sigma Aldrich, St. Louis, MO), which was diluted in phosphate-buffered saline (PBS). The first blood collection occurred prior to treatments and was divided into two equal aliquots: one was incubated with LPS, and the other was incubated with an equivalent volume of PBS. These aliquots were used as positive and negative controls.

At the end of the incubation, all samples were centrifuged at 400 g for 10 minutes to obtain plasma: 250 µl of plasma were mixed with 1000 µl of methanol (1∶5 dilution) to permit protein precipitation. After a final centrifugation at 3000 g for 10 minutes, supernatants were collected and stored at −80°C.

The concentration of plasma PGE_2_ was determined using an enzyme-linked immunosorbent assay kit (Cayman Chemical, Co, Ann Arbor, MI). The calculated coefficient of variation for intra-assay variability was 11.7%, and the inter-assay variability was 9.2%.

#### Cortisol Analysis

Blood for cortisol analysis was collected in a 3-mL heparinized blood collection tube (BD Vacutainer, Franklin Lakes, NJ) and then centrifuged for 10 minutes at 1500 g. The plasma was collected, immediately frozen, and stored at −80°C. Plasma samples were analyzed for cortisol within 60 days after sample collection and within 10 consecutive days once analysis commenced.

Plasma cortisol concentrations were determined using a commercial radioimmunoassay (RIA) kit (Coat-A-Count Cortisol, Siemens Medical Solutions Diagnostics [formally Diagnostic Products Corp.], Los Angeles, CA). Samples were incubated at 4°C for 2 hours to improve assay sensitivity. Samples were assayed in duplicate with the reported concentration equaling the average cortisol concentration between duplicates. The calculated coefficient of variation for intra-assay variability was 9.2%, and the inter-assay variability was 9.3%.

#### Substance P Analysis

Blood (1 mL) for substance P (SP) analysis was collected in a 4-mL potassium ethylenediaminetetraacetic acid (EDTA) purple-top blood collection tube (BD Vacutainer, Franklin Lakes, NJ) that was previously spiked with 50 µL benzamidine. This blood was promptly centrifuged for 15 minutes at 1000 g. The plasma was immediately frozen and stored at −80°C.

The SP assay was performed as described by Liu et al. [14] with slight modifications using non-extracted plasma. Method validation using non-extracted plasma consisted of the complete recovery (±15%) of a known concentration of SP that was added to pooled baseline sample plasma. Samples were analyzed in duplicate with a double-antibody RIA using a primary antibody (polyclonal rabbit anti-SP; 1∶20,000) from Phoenix Pharmaceutical, Inc. (Burlingame, CA, USA). EDTA (13 mM) and benzamidine (1 mM) were added as protease inhibitors. SP was assayed using the ^125^I-[Tyr^8^]-SP tracer (approximately 18000 cpm) (PerkinElmer, Inc., Waltham, MA, USA). Samples were assayed in duplicate with the reported concentration equaling the average substance P concentration between duplicates. The intra- and inter-assay coefficients of variation were 7.6% and 14.9%, respectively.

#### Infrared Thermography Analysis

Standardized anatomical locations on the pig were identified by a technician in IRT digital images that were obtained of study piglets. IRT images were converted to temperature readings by proprietary software that was calibrated internally by the machine and designed to interface specifically with the camera (Thermacam Researcher Pro 2.8 SR-1, FLIR Systems). Data were analyzed for changes in temperature by comparing temperature values obtained at consistent anatomical locations on the pig over the range of sample time points. Four anatomical locations in each image were initially converted to temperature readings, but variations in piglet position and orientation to the camera effectively reduced the sample size for ear and snout readings. Consequently, these were discarded, and the more accessible cranium location was forwarded to the statistical analysis phase of the study.

### Statistical analysis

Data were analyzed using generalized linear mixed models fitted with the GLIMMIX procedure of SAS (SAS Institute Inc., Version 9.2). Treatment, procedure, time, and their interactions were used as fixed effects, whereas sow was a random effect, and piglet was the subject of repeated measures. A separate linear mixed model was run to study the effect of meloxicam concentrations on PGE_2_, substance P and IRT by using Meloxicam_Levels as an explanatory variable. Baseline measurements were used as covariates in the above models. Model assumptions were considered to be appropriately met, based on diagnostics that were conducted on studentized residuals. Estimated least square means and corresponding standard errors, or 95% confidence intervals, are presented. A significant difference was considered to exist when p≤0.05, and a marginal difference was considered to exist when 0.05<p≤0.10. Relevant pairwise comparisons were conducted when the significance of the interaction term was p≤0.10, using Tukey-Kramer adjustments as appropriate to avoid inflation of the Type I error rate due to multiple comparisons.

## Results and Discussion

### Plasma Meloxicam Concentration

**Figure 3:**
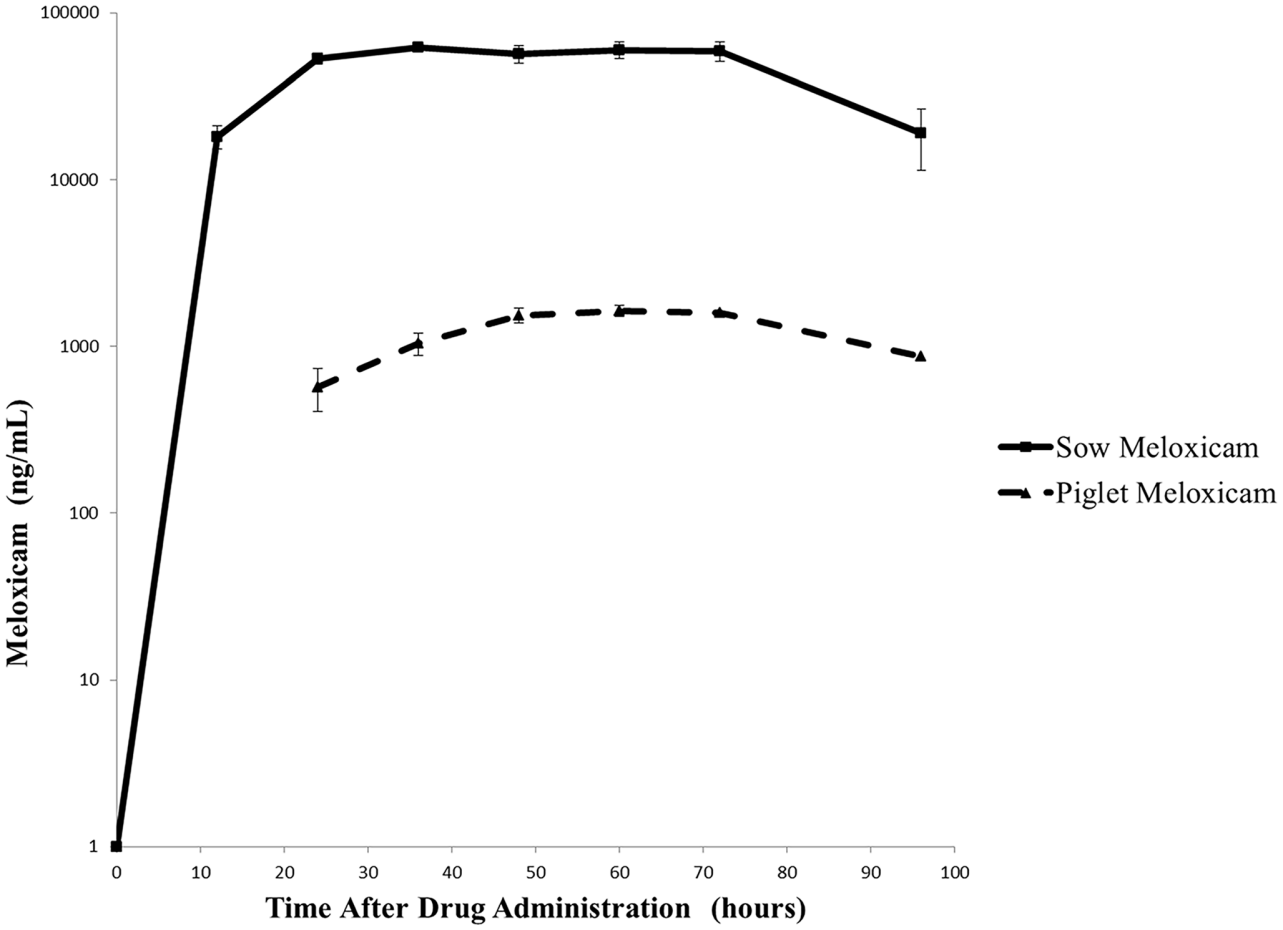
Comparison of plasma meloxicam concentrations from sows and their piglets treated with 30 mg/kg meloxicam. The mean (± SEM) meloxicam levels at 24 hours (piglet processing) were 568.9±105.8 µg/mL. No meloxicam was found in CONT piglet plasma. Both sow and piglet plasma meloxicam concentrations maintained relatively constant levels for the duration of the treatment period, and they began to decline only after 72 hours when the treatment was discontinued in the feed.

Meloxicam was detected in the plasma of all piglets in the MEL group at every time point after treatment commenced (Figure 3). The mean (± standard error of the mean [SEM]) meloxicam concentration at castration was 568.9±105.8 µg/mL. No meloxicam was found in the CONT piglet plasma. Plasma meloxicam concentrations in both sows and piglets maintained steady-state concentrations for the duration of the treatment period, and they began to decline only after 72 hours when the treatment was discontinued in the feed (Figure 3).

The pharmacokinetics of meloxicam after oral administration to mature swine has recently been described [15]. However, this is the first peer-reviewed report of a study documenting the transfer of an NSAID from the sow to the piglet via the transmammary route. In a recent National Pork Board (NPB) report [16], Brown found that injectable meloxicam is transferred through the milk to piglets at processing. However, this transfer utilized a one-time intramuscular dose of 1 mg/kg meloxicam to each sow, which resulted in 2.647 ng/mL of meloxicam in the piglet serum at 5 hours after administration. Piglet plasma meloxicam levels in the NPB report were only measured out to this time point. Due to differences in routes of administration and study design, further comparisons cannot be made. Other species, such as cattle [6] and humans [7], [8], [9], [17], have demonstrated NSAID transfer through milk. The importance of this study was to confirm that the transmammary route of administration is feasible in piglets. Further pharmacokinetic modeling and dose-titration studies are needed to apply this information for the benefit of commercial swine production.

Meloxicam is an NSAID that is approved for swine in Canada and the EU. It is labeled for use in swine to treat non-infectious locomotor disorders by reducing the signs of lameness and inflammation. It is also used for adjunctive therapy in the treatment of puerperal septicemia and toxemia with appropriate antibiotic therapy and for the relief of post-operative pain associated with minor soft tissue surgery, such as castration [18]. Because no analgesic drugs are approved to provide pain relief to swine in the United States, the administration of meloxicam to swine constitutes extra-label drug use (ELDU). Under the Animal Medicinal Drug Use Clarification Act (AMDUCA), ELDU is permitted under veterinary supervision for the relief of suffering in swine when specific conditions are met [19]. In the absence of FDA-approved analgesic compounds in food animals, the use of oral meloxicam tablets for the alleviation of pain or stress in swine can be considered under AMDUCA. It is imperative to remember that the dose of oral meloxicam in this study was extrapolated from data from other species for proof of transfer. Pharmacokinetic analyses are pending and will assist in making further conclusions about the effective dose. However, at this time, the dose used in this study cannot be recommended for use in commercial swine operations due to lack of tissue residue data.

### PGE_2_

**Figure 4:**
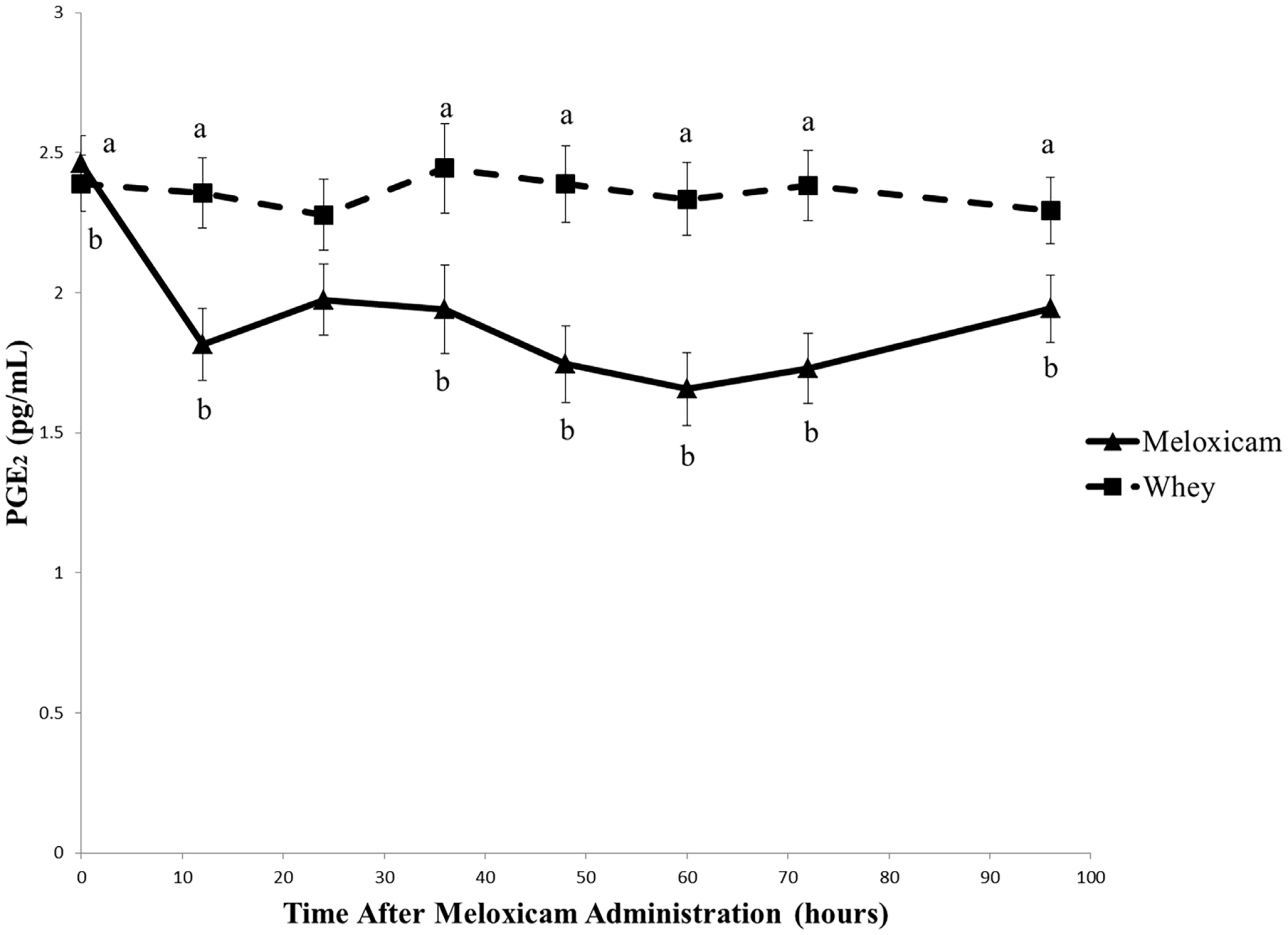
Plasma PGE_2_ ± SE levels from meloxicam (MEL) - and whey placebo (CONT) - treated piglets. MEL piglets had a significantly greater amount of prostaglandin E_2_ (PGE_2_) inhibition compared to their CONT counterparts (p = 0.0059). All time points that are marked with a and b were significantly different (p<0.05). The exception was 24 hours after administration (p = 0.0909).

PGE_2_ demonstrated a treatment effect (p = 0.0059) with significant differences (p<0.05) at each time point, with the exception of 24 hours after drug administration commenced (p = 0.0909) (Figure 4). However, using this analysis, there was no time-by-treatment interaction (p = 0.1763) or effect of time (p = 0.6064). Meloxicam concentration also had evidence of a negative association with plasma PGE_2_ concentrations (p = 0.0048).

This inhibition of PGE_2_ by meloxicam was anticipated, as a result of the blockage of the arachidonic acid pathway and cyclo-oxygenase-2. Prostaglandins contribute to the amplification of pain signaling by increasing nociception sensitization [20]. As such, reducing PGE_2_ concentrations would provide decreased nociception following noxious stimuli, such as castration. Mean piglet plasma PGE_2_ concentrations ranged from 66.2–719.9 pg/mL. These levels are much lower than those reported in equines (1.7 ng/mL), canines (329 ng/mL), and felines (0.7 ng/mL) [21]. No porcine comparisons are available in the literature. There are several potential explanations for the lower PGE_2_ levels. First, the piglets were relatively young and blood from these animals may not have fully responded to LPS stimulation. Second, other studies have used different strains or concentrations of LPS. Finally, we used *ex vivo* stimulation of whole blood, which may contribute to lower levels. Despite these species-specific differences, decreases in plasma PGE_2_ were observed at most time points in MEL piglets compared to CONT piglets. This suggests that meloxicam was successfully transferred through milk to piglets at concentrations that likely provided analgesia based on the demonstrated ex-vivo inhibition of PGE_2_ production.

### Pain Biomarker Analysis

#### Cortisol Analysis

**Figure 5:**
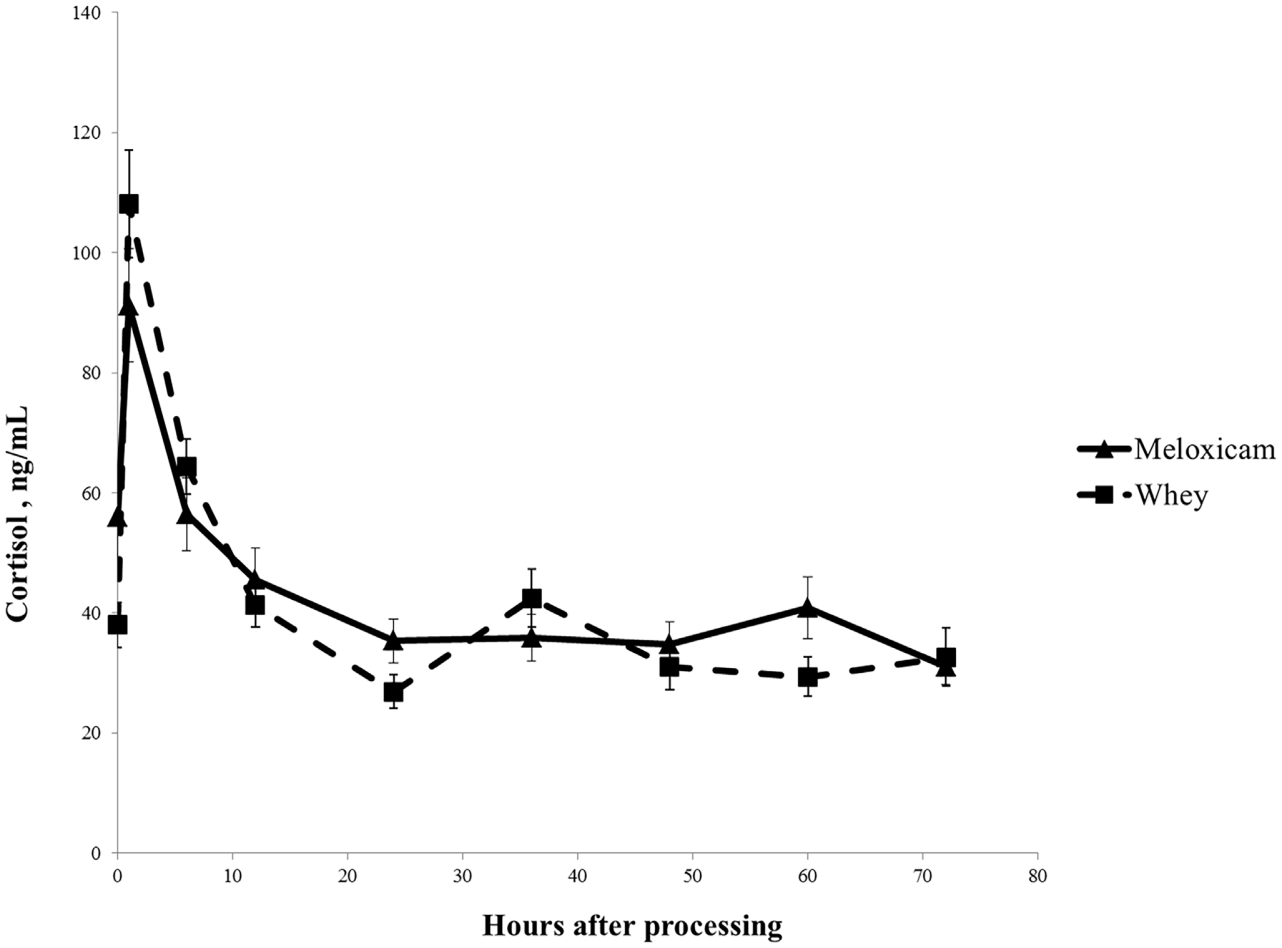
Processed plasma cortisol concentrations after the treatment of sows with 30 mg/kg meloxicam (MEL) or whey placebo (CONT). Means ± SE are depicted.

There was a time-by-treatment interaction for piglet plasma cortisol (p = 0.0009) (Figure 5). MEL piglets had lower plasma cortisol than CONT piglets for the first 10 hours after processing. Although no individual time points demonstrated significant differences, p values with marginal significance were observed at 1 and 6 hours after processing (p = 0.10 and p = 0.12, respectively; (Table 1).

**Table 1:** Mean plasma cortisol concentrations (± SEM) after processing in piglets treated with meloxicam (MEL) or whey (WHEY) placebo.

Time	MEL	WHEY	p
	Plasma Cortisol (ng/mL)	Plasma Cortisol (ng/mL)	
1 h	91.18±9.43	108.15±8.96	0.12
6 h	56.46±6.07	64.36±4.63	0.10
12 h	45.45±5.29	41.30±3.65	0.74
24 h	35.38±3.65	26.86±2.79	0.37
36 h	35.86±3.90	42.49±4.85	0.15
48 h	34.83±3.68	30.99±3.75	0.78
60 h	40.86±5.15	29.36±3.27	0.45
72 h	30.98±2.92	32.66±4.80	0.73

These study findings are in agreement with several other studies that associate a decrease in piglet plasma cortisol with pain mitigation. This decrease was noted with various analgesics at castration, such as meloxicam [22], [3], both meloxicam and flunixin administered separately [23],[24], and both meloxicam and tolfenamic acid administered separately [4].

The highest cortisol levels in both groups were observed at 60 minutes after castration. This peak is shown in Figure 5 and is consistent with previous studies showing that the highest cortisol levels are detected 30–90 minutes after processing [22], [25], [4], [23], [26], [27], [28].

Glucocorticoids are secreted in response to a stressor, such as castration, and are generally considered to be indicative of stress and, thereby, pain. [27]. However, piglet plasma cortisol does have some limitations as an objective pain biomarker. For instance, stressors such as handling may cause an increase in plasma cortisol concentrations [29]. However, research by Prunier et al. [28] suggests that sham-castrated pigs have lower amplitudes and durations of cortisol than castrated piglets, and these are likely to be connected to pain or tissue damage [22]. A systematic review of pain management during routine management procedures highlighted the need for additional validation of pain biomarkers in peer-reviewed studies [30], [31]. Until more pain biomarkers can be clearly described and validated, cortisol remains to be one of the most easily identifiable means of describing piglet pain.

#### Substance P

Measurements of SP indicated no differences between MEL and CONT piglets at processing (p = 0.6733). There was a significant change in SP levels over time (p = 0.0024). However, there were no significant interactions in procedure by time (p = 0.66) or treatment by procedure by time (p = 0.33) (Table 2). There was also no association between meloxicam and SP levels (p = 0.1444).

**Table 2:** Comparison between the least squares (LS) means ± standard error (SE) of piglet serum chemistry biomarkers and infrared thermography (IRT) temperatures, as classified by the procedure (Proc) of castrated (CAST) and sham castrated (SHAM) and treatment (Trt) with 30 mg/kg PO meloxicam (MEL) or whey placebo (CONT) to sows on Days 4–6 after farrowing.

Proc	Experimental Group Calculated Means (± SEM)				P VALUES				
	CAST		SHAM		(model adjusted)				
Trt	CONT	MEL	CONT	MEL					
**Parameter**	LS Means± SE	LS Means± SE	LS Means± SE	LS Means± SE	Time	Trt	TimeXTrt	ProcXTime	TrtXProcXTime
**Average Cortisol, ng/mL**	48.9±3.49	50.41±3.82	43.71±2.96	44.53±2.73	<0.0001	0.65	0.0009	0.14	0.39
**Average Substance P, pg/mL**	89.24±4.34	95.59±2.54	81.13±3.52	96.60±3.48	0.0024	0.35	0.67	0.66	0.33
**Left Ear Temp, 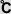 **	32.06±0.30	32.42±0.26	32.56±0.30	32.35±0.26	<0.0001	0.85	0.97	0.73	0.96
**Right Ear Temp, 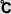 **	34.37±0.22	33.80±0.23	34.07±0.22	33.85±0.21	<0.0001	0.69	0.80	0.58	0.74
**Snout Temp, 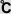 **	31.56±0.25	32.40±0.23	32.10±0.25	31.93±0.25	<0.0001	0.79	0.09	0.63	0.49
**Cranium Temp, 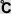 **	37.35±0.10	37.55±0.08	37.35±0.09	37.47±0.08	<0.0001	0.32	0.01	0.87	0.99

SP is an 11-amino acid neuropeptide that regulates nocioreceptive neurons, which are involved in the integration of pain, stress, and anxiety [32], [33]. It has proinflammatory effects in immune and epithelial cells and participates in inflammatory diseases of the respiratory, gastrointestinal, and musculoskeletal systems [34].

These results are in agreement with a recent study by Sutherland et al. [25], in which no significant differences in SP levels between castrated and sham-castrated piglets were found. However, in bovines, Coetzee et al. [33] demonstrated that castrated calves have significantly elevated SP levels compared to their non-castrated counterparts. Although SP has the potential to accurately describe physiological pain, further research is needed to determine its value in swine.

#### Infrared Thermography

**Figure 6:**
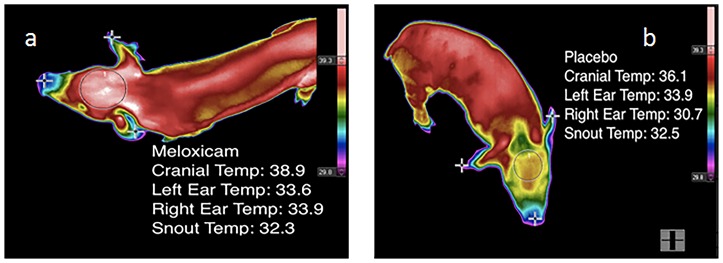
Example IRT images from a meloxicam-treated (a) and placebo-treated control piglet (b) after castration. Color differences reflect activation of the sympathetic nervous system leading to peripheral vasoconstriction and a localized decrease in skin temperature. Figure 6a demonstrates a meloxicam-treated piglet with a higher (red) cranial skin temperature than the cranial skin temperature (yellow) of the whey-treated piglet.

**Figure 7:**
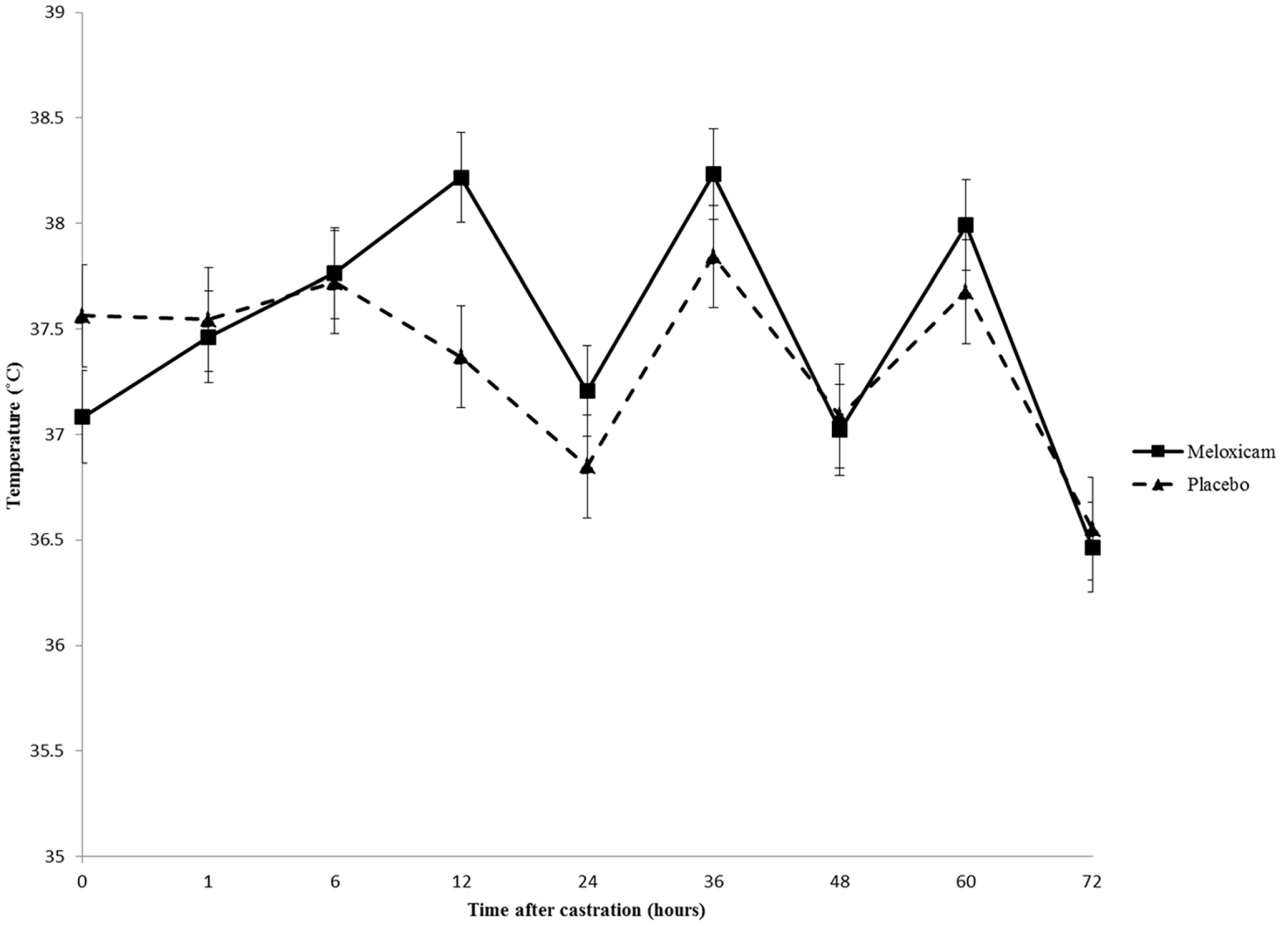
Cranial infrared thermography (IRT) from meloxicam (MEL)- and whey placebo (CONT)- treated piglets. Means ± SE are depicted. There is a significant time-by-treatment interaction between MEL and CONT piglets (p = 0.0148). The interaction was significant at all timepoints (p<0.0001). There was an association between plasma meloxicam levels and cranial IRT measures (p = 0.0345).

Example IRT images from a meloxicam-treated and placebo-treated control piglet after castration is presented in Figure 6 a-b. IRT demonstrated a significant time-by-treatment interaction in cranial temperature between MEL and CONT piglets (p = 0.0148; Figure 7). The interaction was significant at all timepoints after castration (p<0.0001; Figure 7). After baseline measurements, CONT piglets had lower skin temperature than MEL piglets. There was a positive association between plasma meloxicam levels and cranial skin temperature (p = 0.0345).

Animals that are stressed or in pain can exhibit decreases in cutaneous temperature due to sympathetic nervous system activation, which causes vasoconstriction, shifting of the blood from the skin to the organs, and loss of heat in the periphery of the body [35], [36]. IRT has been shown to be a valuable tool for pain assessment in beef and dairy calves by non-invasively measuring autonomic nervous system responses at the time of dehorning and castration [36], [35], [37]. IRT was also used by Hansson et al. [24] to measure temperatures at 24 hours after piglet castration. Piglet ear temperature was found to be significantly higher in control piglets versus those given either lidocaine or a lidocaine/meloxicam combination. In that same study, no significant differences were found when measuring the skin around the castration site. This one-time measurement reflects the differences noted in this study up to 24 hours after castration (Figure 6) but fails to provide a longer duration depiction of piglet pain.

Differences in IRT measurements in anatomical sites were noted. Cranial temperature was lower in CONT piglets. This significant time-by-treatment interaction (p = 0.0148) suggests that this would be an effective anatomical site for assessing the effect of pain on cutaneous perfusion However, there were no significant differences in temperature between treatment groups over time in the left ear (p = 0.9744), right ear (p = 0.7989), and snout tip (p = 0.0936). There was also no association between plasma meloxicam concentrations and IRT measurements in the snout (p = 0.8683), left ear (p = 0.9141), and right ear (p = 0.2029) (Table 2).

Analysis of the ears and snout areas proved to be difficult, due to the image capture method. Thermography images were taken by placing each piglet in a small plastic tub to reduce any confounding stress that was associated with further handling after castration and blood sampling. It was challenging to obtain consistent images, due to the anatomical configuration of the folded-over, floppy ears on a relatively mobile piglet. Also, the snout may have been too sensitive to ambient temperature to provide a meaningful assessment of individual piglet pain. Therefore, for the purposes of this study, it was determined that cranial skin temperature was the most accurate anatomical location for assessing piglet pain responses after castration.

The temperature measurement sites that were found to be useful in this study are in conflict with other studies using IRT. Schmidt et al. [38] found the eye and the back of the ear to be the most useful for assessing fever in sows. Additional sites in the literature include the mammary gland and vulva [39], [40]. However, these studies detected either fever response or estrus onset in adult animals, which are likely different than pain-related thermoregulation processes in baby piglets.

Temperature differences in the treatment groups are further accentuated in a circadian rhythm. Peak temperatures in both MEL and CONT piglets were noted in the evening measurements at 12, 36, and 60 hours after castration. Trough temperatures were seen in morning measurements at 24, 48, and 96 hours after castration (Figure 6). Similar temperature circadian trends have long been noted in livestock, and they were recently demonstrated in dairy cows [41].

Measurement of piglet body temperature using IRT shows promise as a piglet pain biomarker by demonstrating differences in cranial temperature. This non-invasive method allows pain to be assessed for up to 72 hours after castration.

This study is the first peer-reviewed report of the successful transmammary transfer of meloxicam in milk from sows to piglets. Piglet plasma cortisol levels and cranial IRT measurements demonstrated significant changes as a result of analgesic treatment with meloxicam. The novel administration of analgesic drugs via transmammary transfer has significant potential benefits for the swine industry. As one litter is medicated through the oral treatment of one sow, large numbers of piglets can receive pre-emptive analgesia without the need for additional handling and injections. This will also lead to reduced animal stress, improved safety for both the pig and handler, and a reduced potential for tissue lesions and drug residues when the injections are removed. Future research investigations can focus on providing data for meloxicam dose refinement and validating physiological pain indicators.
